# Rational Protein Engineering to Increase the Activity and Stability of *Is*PETase Using the PROSS Algorithm

**DOI:** 10.3390/polym13223884

**Published:** 2021-11-10

**Authors:** Andrew Rennison, Jakob R. Winther, Cristiano Varrone

**Affiliations:** 1 Section for Biomolecular Sciences, Linderstrøm-Lang Centre for Protein Science, Department of Biology, University of Copenhagen, Ole Maaloes Vej 5, 2200 Copenhagen, Denmark; Andrewrennison100@gmail.com; 2 Section for Sustainable Biotechnology, Department of Chemistry and BioScience, Aalborg University, A.C. Meyers Vænge 15, C2, 2450 Copenhagen, Denmark

**Keywords:** PETase, thermostability, thermal deactivation assay, PET hydrolysis

## Abstract

Polyethylene terephthalate (PET) is the most widely used polyester plastic, with applications in the textile and packaging industry. Currently, re-moulding is the main path for PET recycling, but this eventually leads to an unsustainable loss of quality; thus, other means of recycling are required. Enzymatic hydrolysis offers the possibility of monomer formation under mild conditions and opens up alternative and infinite recycling paths. Here, *Is*PETase, derived from the bacterium *Ideonella sakaiensis*, is considered to be the most active enzyme for PET degradation under mild conditions, and although several studies have demonstrated improvements to both the stability and activity of this enzyme, stability at even moderate temperatures is still an issue. In the present study, we have used sequence and structure-based bioinformatic tools to identify mutations to increase the thermal stability of the enzyme so as to increase PET degradation activity during extended hydrolysis reactions. We found that amino acid substitution S136E showed significant increases to activity and stability. S136E is a previously unreported variant that led to a 3.3-fold increase in activity relative to wild type.

## 1. Introduction

Polyethylene terephthalate (PET) is a synthetic aromatic polyester and is one of the most commonly used materials for textiles and (food and drinks) packaging and is appreciated for its high durability, low weight, and gas permeability as well as its resistance to degradation [1]. With up to 580 billion PET bottles predicted to be produced globally throughout 2021 [2], it is clear that the material plays a large role, both economically and environmentally. The most commonly used recycling process comprises sorting, mechanical rinsing and grinding, followed by melt processing and extrusion. These processes reduce the chain length and crystallinity of PET fibres, finally resulting in reduced product quality [1,3]. Additionally, while recycling is a top priority, in 2018, only approximately 40% of plastic packaging waste was recycled in the EU, with the majority of the rest going to landfills or being incinerated [4]. Furthermore, much of the virgin PET produced from petroleum resources is lost as waste (incinerated or landfilled) and is unable to be recycled and reused in its most valuable form. Thus, in order to ensure that this useful material is kept within a closed loop, according to the circular economy concept, a recycling method that can depolymerise post-consumer PET (PC-PET) into monomers, which can be repolymerised into high crystallinity PET, is required.

PET is made of repeating units of mono(2-hydroxyethyl) terephthalate (MHET) that are linked by ester bonds. Many bacterial and fungal enzymes have been identified that can convert PET into this monomer [5]. MHET, which is also an ester, can also be further hydrolysed into its constituents ethylene glycol (EG) and terephthalic acid (TPA). These PET-degrading enzymes are often lipases or cutinases, yet they often have relatively low turnover rates at the low temperatures that would be required for an economically viable recycling process [3]. In 2016, interest in PET-degrading enzymes was raised by the discovery of a bacterium, *Ideonella sakaiensis*, in a bottle recycling facility in Japan that could grow solely on PET as an energy and carbon source. An enzyme of the cutinase family, designated as *Ideonella sakaiensis* PET hydrolase (*Is*PETase), was identified in this bacterium that could depolymerise PET together with the action of a second enzyme called mono(2-hydroxyethyl) terephthalate hydrolase (MHETase) [6]. *Is*PETase demonstrated higher PET degradation activity compared to other enzymes known at the time and, crucially, was active in mesophilic conditions, with an optimum temperature of 30 °C [6,7]. This seemed to indicate the potential applicability of the enzyme in biotechnological recycling and upcycling. However, *Is*PETase was much less thermally stable than other known PET-degrading enzymes, causing issues with expression yields and extended processing times. Although the enzyme had the highest known activity at low temperatures [6], it still did not have the required activity or stability to form part of an economically viable depolymerisation process [8].

*Is*PETase has an alpha/beta hydrolase fold and uses a serine hydrolase mechanism that is similar to trypsin. It is not known to what extent *Is*PETase has evolved by natural selection in *I. sakaiensis*, but as PET has only been found at significant levels in nature for about 50 years, it is likely that the enzyme has not yet reached its full evolutionary potential. Thus, several studies have been performed to increase the activity and thermostability of the enzyme through rationally designed point mutations [3]. Examples of improved variants include ^Austin^PETase [9] and ^Joo^PETase [10], which were both produced to improve activity, and ^Son^PETase [11], which developed with the goal of increasing stability. ^Austin^PETase was devised by applying substrate docking calculations to suggest mutations and to narrow the active site cleft by introducing the substitutions W159H and S238F. ^Joo^PETase was obtained in a similar manner, resulting in R280A, which removed a protruding arginine residue from the substrate binding pocket. Finally, ^Son^PETase was built on the R280A variant by adding a further two substitutions, S121E and D186H, to stabilise the loop regions.

In this study, a computational approach was used in which amino acid substitutions were recommended for the stabilisation of the enzyme by the Protein Repair One Stop Shop (PROSS) method. These were visually examined in a molecular viewer (PyMol) to determine their suitability. Variants were then experimentally assessed based upon their thermostability by differential scanning fluorimetry (DSF) and a thermal deactivation assay. Activity was also assayed on standardised PET films over a range of temperatures.

## 2. Materials and Methods

### 2.1. PROSS

The online PROSS server at (https://pross.weizmann.ac.il/step/pross-terms/ accessed: 12 Feburary 2020) was used for the design of the mutations that could be used to increase the thermostability of *Is*PETase. The crystal structure of *Is*PETase was taken from [10] (PDB code: 5XJH). The residues forming the active site, the oxyanion hole, and the binding residue at W185 were fixed during the mutation design, preventing them from being altered. The standard multiple sequence alignment (MSA) parameters for the PROSS server were used. This included only allowing proteins with an identity of over 35% and with a coverage of over 75%, both taken from a BLAST search, to be included in the alignments. The maximum number of sequences to be used in the alignment was 4000, and the energy function used in the Rosetta calculations was talaris 2014.

### 2.2. Plasmid Construction

The base plasmid used for the expression of all of the variants was pET21b(+)-Is-PETase, obtained from AddGene (plasmid #112202) [9]. The PETase sequence was obtained from [9], following which the gene was codon optimised for expression in *E. coli* and was cloned into the expression vector pET-21b(+) with a C-terminal His tag. The endogenous signal peptide was removed from the gene by polymerase chain reaction (PCR) and subsequent kinase, ligase, DpnI (KLD) treatment, as described below.

### 2.3. Variant Construction

Mutations were introduced to the wild-type gene by PCR with mutagenic primers, which were designed using the online tool NEBasechanger, which is available at (https://nebasechanger.neb.com/ accessed: 16 January 2020). Codons for the new amino acids that were to be introduced were chosen from the codon usage chart in *E. coli* K12 genes [12]. Following the introduction of the point mutations by PCR, the linear mutated fragments were circularised using the KLD kit (NEB), which included a DpnI enzyme to remove any template DNA. The reaction mixture was incubated at room temperature for 10 min before being directly used in the transformation. The gene for ^Son^PETase was purchased from Twist Bioscience (San Francisco, CA, USA) and was introduced into the pET-21b(+) backbone by Gibson assembly using standard protocols. All of the primers used for cloning and site-directed mutagenesis are listed in S1.

### 2.4. Protein Expression and Purification

Single clones of *E.* *coli* BL21(DE3) cells containing relevant plasmids were inoculated into 10 mL of LB medium with 100 µg/L of ampicillin at 37 °C at 200 rpm. The overnight culture was then inoculated into a volume of 500 mL LB-AB medium in 3 L flasks, with an initial growth temperature of 37 °C, and the samples were shaken at 200 rpm. After the culture reached OD_600_ of 0.4–0.6, the temperature was then reduced to 16 °C, and expression was induced with 0.4 mM isopropyl β-D-1-thiogalactopyranoside (IPTG), and the culture was allowed to grow for 24 h. The cells were harvested by centrifugation for 15 min at 7000 rpm and 4 °C, the supernatant was discarded, and the pellet was frozen at −20 °C. The pellets were resuspended in 20 mL of lysis buffer (50 mM phosphate buffer pH 7.0) and were lysed by ultrasonication in two rounds of 8 × 30 s pulses, with the mixture kept on ice. This mixture was then centrifuged at 18,000 rpm and 4 °C for 15 min, and the supernatant was retained. His-pur Ni-NTA agarose resin (Thermo-scientific, Roskilde, Denmark) was equilibrated in lysis buffer on a gravity flow column (Bio-rad, Hercules, CA, USA) ensuring a column volume of approximately 2 mL for each 20 mL of cell lysate. The resin was then resuspended in approximately 10 mL of the phosphate buffer and was mixed with cell lysate at 4 °C overnight to allow the His-tagged proteins to bind to the Ni-NTA resin. Following this, the lysate/bound resin mixture was again added to the gravity flow column and was eluted with increasing concentrations of imidazole in 50 mM phosphate buffer at pH 7.0, with the target protein eluting at 250 mM imidazole. The eluted fraction was subjected to dialysis in 3.5 kDa molecular weight cut-off (MWCO) standard regenerated cellulose (RC) dialysis tubing (Spectrum labs) to wash out the imidazole in the dialysis buffer, and afterwards, it was stored in 50 mM phosphate buffer with a pH of 7.0. Protein concentration was determined at 280 nm using an extinction coefficient of 39,420 cm^−^^1^M^−^^1^.

### 2.5. Assessment of Melting Temperature by Differential Scanning Fluorimetry

The T_m_ of variants was determined by DSF on a Prometheus (NanoTemper Technologies GmBH, München, Germany) according the manufactures directions. Accordingly, protein fluorescence was monitored at 330 nm and 350 nm in lysis buffer at a protein concentration of ~10 µM while the temperature increased from 20 °C to 90 °C at 0.5 °C per min. T_m_ was determined at the inflection point of the second derivative of the E_330_/E_350_-ratio.

### 2.6. Assessment of Melting Temperatures by Thermal Deactivation Assay

The activity of *Is*PETase on PET can be defined as an ester hydrolysis, and as such, a medium-throughput assay was developed to quantify the reduction in the activity of the enzyme during incubation at a specific temperature over time. We used para-nitrophenyl acetate (pNPA) (Thermo-scientific, Roskilde, Denmark) as a model esterase substrate [13]. Purified enzyme was standardized to 5 μM in 500 μL of lysis buffer and was incubated at 45 °C for various time periods. At each timepoint, 20 μL of sample was removed from the incubation tube and was added to 170 μL of phosphate buffer in a 96-well plate kept on ice. After the incubation period, the plate was allowed to come to room temperature, and 10 μL of 20 mM *p*NPA in dimethyl sulfoxide (DMSO) (Thermo-scientific, Roskilde, Denmark) was added to each well using a multichannel pipette. The plate was then incubated at 25 °C in a spectrophotometer, and the absorbance was measured at 405 nm at 1 min intervals. All of the experiments were done in triplicate, and a blank control assay was conducted with the phosphate buffer replacing the enzyme. The linear range of the assay with respect to product *p*-nitrophenol was determined, and only absorbance values that were within this range were accepted. The slope up to the first five points on the curve of absorbance against time was then calculated for each incubation time, and this was then taken as the initial activity. The slope of the blank control assay was used to correct activity measurements for background hydrolysis. The initial rate of reaction was then plotted against incubation time in order to determine the rate of denaturation and the half-life (*t*_1/2_) of the enzyme following incubation. Due to the large error resulting from background correction, any activities that were less than 5% of the initial activity or less than double that of the blank control assay were discarded. Finally, only decay curves with three or more points satisfying these data acceptance criteria were accepted. The *t*_1/2_ was calculated by fitting the data to conventional exponential decay, as expressed by Equation (1), where *A_t_* is the activity at time *t*, *A*_0_ is the initial activity, and *k* is the rate constant for decay:

(1) $$A_{t}=A_{0}\;e^{-kt}$$

The half-life of the decay of the activity of each variant upon incubation was then calculated using Equation (2), where *t*_1/2_ is the half-life, and *k* is the rate constant for decay:

(2) $$t_{1/2}=\frac{Ln\left(2\right)}{k}$$

### 2.7. Assessment of PET Hydrolysis

For simplicity, we chose an assay modified from [14], where standardised and commercially available PET film (Goodfellow, UK) was the substrate and the release of MHET/TPA was detected at 260 nm using an NanoDrop 1000 spectrophotometer (Thermo-scientific, Roskilde, Denmark). PET discs were cut from a 0.25 mm thick amorphous PET sheet using a standard office hole punch, creating ¼″ discs with a calculated surface area of 0.64 cm^2^. The discs were submerged in ethanol and were allowed to dry in a laminar flow bench in order to sterilise them. They were then placed in a 2 mL eppendorf tube with 100 nM of enzyme in a 50 mM tricine pH 9.0 buffer with 10% (*v*/*v*) DMSO for a total reaction volume of 250 μL. Short Pasteur pipette lengths made from polyethylene were placed on top of the discs to ensure that they did not float on top of the small volume of liquid due to the high surface tension. Samples were shaken (200 rpm) at various temperatures in a shaking incubator (IKA KS 3000i) (Staufen, Germany). Samples of 2 μL in volume were taken at regular intervals, and the absorbance was read at 260 nm. Standard curves made using the high-performance liquid chromatography (HPLC) method described below gave extinction coefficients for MHET and TPA, respectively, as 135.4 M^−^^1^cm^−^^1^ and 118.4 M^−^^1^cm^−^^1^. Samples of the hydrolysis products also showed an MHET concentration that was approximately 5 times higher than that of TPA after 24 h on average. For simplicity, and since the extinction coefficients are similar, the product is reported as the MHET equivalents, i.e., the sum of TPA and MHET.

### 2.8. High Performance Liquid Chromatography Analysis

The method was modified from the one reported by [15]. Samples with a volume of 50 μL were taken and centrifuged at 20,000 rpm for 10 min, and the supernatant was diluted in a 1:1 ratio with methanol. Standards of TPA (Thermo-scientific, Roskilde, Denmark), MHET (Advance ChemBlock Inc., Burlingame, CA, USA), and Bis(2-hydroxyethyl) terephthalate (BHET) (Thermo-scientific, Roskilde, Denmark) taken also made in a 50% methanol solution (*v/v*) to ensure equivalence with the samples from 0.05 mg/mL to 25 mg/mL. Any concentrations below this range would be considered below the method’s limit of quantification. The samples were analysed using a Dionex Ultimate 3000 system (Sunnyvale, CA, USA) fitted with a UV/Vis detector. A C18 column (Phenomenex Luna 5 μm, 250 mm × 4.6 mm) (Torrance, CA, USA) at 35 °C was used for the separation of the reaction products, with a mixture of 60% Milli-Q water, 20% acetonitrile, and 20% 10 mM H2SO4 (*v/v/v*) being used as the mobile phase. The flow rate was 1 mL/min, and the injection volume was 10 μL, with detection at 260 nm.

### 2.9. Kinetic Analysis of Variant Hydrolysis of PET

Inverse Michaelis–Menten (^Inv^MM) determinations were based on the methods outlined in [16]. Amorphous PET discs produced in the same method as described above were added to a variety of enzyme concentrations between 50 nM and 650 nM, with the amount of degradation products quantified as described above. The linearity of the progress of the reaction over time was confirmed, and final samples were taken after 6 h of hydrolysis. Rates were then reported in units of nmol_Products_min^−^^1^, where ^Inv^V_max_ is the maximum rate in the units above, as determined by the ^Inv^MM kinetics as described by [16]. Calculations of ^Inv^V_max_ and ^Inv^K_m_ were performed using the Prism (GraphPad, San Diego, CA, USA) statistical software by fitting the data to a standard Michaelis–Menten equation. Confidence intervals of 95% were also determined using this software.

## 3. Results and Discussion

### 3.1. Protein Engineering to Improve Ths Stability of IsPETase Using PROSS

The upload of the structure for IsPETase (PDB ID: 5XJH) into the PROSS server resulted in an extensive list of 51 substitutions, see Supplementary Information for the full list (S2). As this number was too large to be studied in the scope of this project, a limited few were selected to be studied on the basis of their alignments to three highly efficient thermostable PET hydrolysing enzymes, namely Cutinase 2 from Thermobifida fusca (TfCut2) [17], an Alpha/Beta hydrolase family protein from Saccharomonospora viridis (Cut190) [18], and the Leaf Compost Cutinase from an uncultured organism (LCC) [19], and were discarded if no agreements were found. From this process, three novel variants were selected for experimental characterization: ^S125R^PETase, ^S136E^PETase, and ^T270Q^PETase. The alignments can be seen in Table 1, and the visualization of the substitutions can be found in the Supplementary information (S3).

From this series of alignments, we can see that the enzyme Cut190 contains the relevant amino acid corresponding to the substitutions designed by the PROSS server. As this enzyme is active on PET and since it has a higher thermostability than IsPETase, we surmised that the substitutions at these positions would at least have no deleterious effects and would therefore be studied through further experiments.

### 3.2. Improved Thermostability of IsPETase Variants

Variants were constructed in plasmids containing a truncated *Is*PETase gene with the *I. sakaiensis* signal peptide removed using PCR with mutagenic primers. The protein was produced intracellularly and was purified to over 85% purity, as described in the Materials and Methods section.

**Figure 1:**
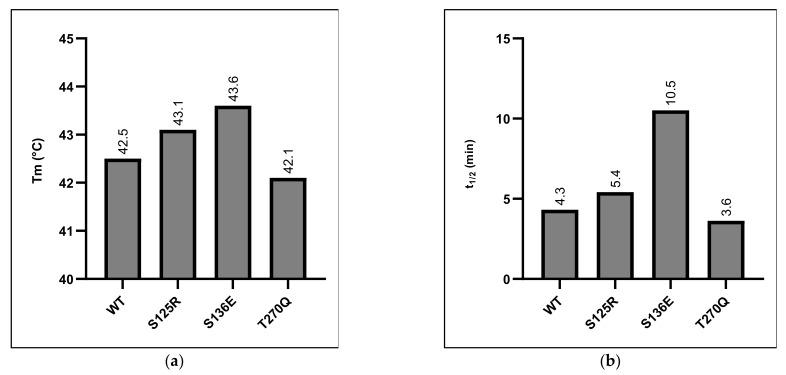
Determination of thermostability of PETase variants and wild-type enzyme. Panel (**a**): Dye-free differential scanning fluorimetry measuring the ratio between fluorescence of tryptophan residues in a folded and unfolded state. Panel (**b**): Half-lives of enzyme activity on the pNPA model substrate following incubation at 45 °C.

To examine the effect that the rationally designed substitutions had on thermostability, we used two assays: a structure-based heat inactivation and an activity-based assay that was developed during the study. In the second assay, the activity half-life was determined to create a better picture of how the protein behaves under typical hydrolysis conditions, with any partial unfolding leading to partially active enzyme or refolding upon cooling. Upon the incubation of both the variant and the wild-type enzymes at 45 °C, the samples were withdrawn for activity determination using a para-nitrophenol acetate (pNPA) substrate. From these measurements, activity half-lives were determined. Although this assay measured the hydrolysis of a small model substrate, we anticipate that it mimics the inactivation of *Is*PETase at this temperature. The difference in pH between this inactivation and the PET hydrolysis experiments is not expected to make differences in the relative stabilities between variants. Thus, when assaying PETase activity, it is likely that relative differences between the half-lives of variants (during PET hydrolysis at increased temperatures) will be similar to those seen here. Results from these stability assays are shown in Figure 1, with progress curves and decay curves resulting from the thermal deactivation assay in the Supplementary information (S4).

The T_m_ of *Is*PETase reported here, at 42.5 °C, is somewhat lower than that found in other studies, where values of 50.8 °C [20], 48.8 °C [10], and 46.8 °C [9] have been reported when using dye-based approaches at various pH levels. However, with wild-type *Is*PETase as reference, we found that ΔT_m_ is the highest in ^S136E^PETase (1.1 °C), with all of the other variants showing differences of less than a degree compared to the wild-type enzyme (Figure 1a). As these differences are rather small compared to the overall T_m_, in order to confirm that this was a genuinely improved variant, we also tested all of the enzymes with the thermal deactivation assay. The pattern of the differences in t_1/2_ compared to the wild-type enzyme is broadly the same as the pattern seen in the Tm determinations. The activity half-life, t_1/2_, of the ^S136E^PETase variant displays a 2.4-fold increase when measured at 45°C, suggesting that this variant is actually improved in terms of thermostability when compared to *Is*PETase (Figure 1b).

The relative increases of thermostability seem to be somewhat different between the two assays, with t_1/2_ showing a much larger increase than T_m_. However, the direct comparison of these two related variables is difficult, as they essentially measure different properties. This does suggest some that some interesting mechanisms are at play during the loss of activity in the ^S136E^PETase variant. As the enzyme samples were cooled on ice after incubation, it could be suggested that some measure of refolding into an active conformation occurs following incubation at 45 °C. This could lead to an apparent increase in the thermostability as measured by t_1/2_, which would not be discernible with the measure of T_m_. Refolding in this manner could lead to several mechanisms to retain activity, such as the prevention of aggregate formation or the reassembly of local structures that allow partial activity to be retained. The measure of t_1/2_ could therefore result in a more illustrative measure of stability that applies better to the real-world applications of these enzymes.

Taking these two sets of results together, we concluded that only ^S136E^PETase showed a consistent improvement in terms of thermostability. Therefore, it was decided that ^S136E^PETase would be further assayed for activity on a PET discover a range of temperatures compared to the wild-type enzyme.

### 3.3. Increased PET Degradation Activity of ^S136E^PETase

**Figure 2:**
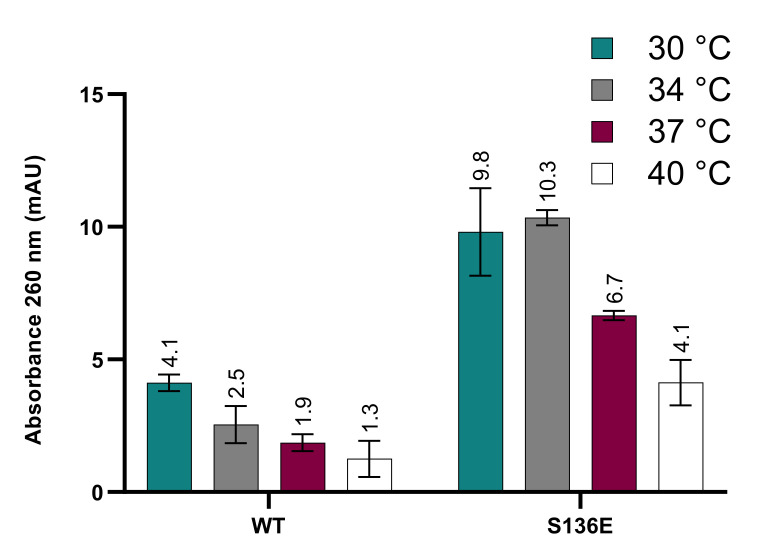
Hydrolysis rates of standardized PET discs over 24 h by ^S136E^PETase variant and wild-type enzyme at a concentration of 100 nM over a range of relevant temperatures. Error bars represent the standard deviation of triplicate measurements. Absorbances are based on a path length of 1 cm.

As stated above, comparison between various studies reporting increased IsPETase variant activity can be difficult due to the absence of a standardised PET hydrolysis activity assay and the different types/crystallinity of the PET substrates that are used. Unfortunately, it is often difficult to distinguish the effects of PETase variants over extended time-courses, as thermostability and activity become intertwined when the half-life and assay time are of a comparable magnitude. Thus, apparent increases in activity may be indistinguishable from (or at least masked by) higher stability, especially at higher temperatures. Nevertheless, as IsPETase did not produce a quantifiable amount of degradation product over shorter timescales, it was necessary to assay over a longer period. The results of this PET degradation assay for ^S136E^PETase compared to IsPETase are shown in Figure 2, with ^S125R^PETase, and ^T270Q^PETase being omitted due to their marginal differences in thermostability, as stated above.

The data above show that *Is*PETase, as expected, shows a significant loss of product formation as the temperature is increased from 30 °C to 40 °C. ^S136E^PETase seems to show a higher thermostability in this assay also, with a relative reduction in product formation at higher temperatures that is smaller than that seen in the wild-type enzyme. Notably, ^S136E^PETase shows a significant increase in product formation compared to *Is*PETase, with 3.3 times higher absorbances at the optimum temperature of 34 °C. This increase compares favourably with the activity increases seen for other variants [10,20]. While it is somewhat surprising to find a mutation that leads to increases in both thermostability and activity, it appears to be the case here. However, there are complex factors that take place in the hydrolysis of an insoluble substrate, such as changes to the crystallinity as the plastic moves towards the glass transition temperature, or the higher motility of the termini of polymer chains at higher temperatures. As such, it cannot be expected that the simple trade-off between stability and activity be borne out in this case.

### 3.4. Kinetic Analysis of ^S136E^PETase Variant

As already mentioned, it is difficult to decouple the effects of thermostability and intrinsic activity in assays of PETase variants. Given the low activity and stability of the wild-type enzyme, it was not possible to measure the degradation products during the linear part of the progress curve for the hydrolysis. Therefore, we decided to remove any effects of thermal degradation during the assay of the variants and to compare ^S136E^PETase to a variant that was previously shown to have a higher stability ^Son^PETase [11]. Unfortunately, conventional Michaelis–Menten kinetics can only be applied to conditions where substrate concentrations can be varied homogeneously in solution. With a solid substrate such as PET, this is, of course, not possible, and for this reason, an alternative approach to kinetic characterization, termed ^Inv^MM kinetics, has been recently developed [15,21]. The ^Inv^MM framework relies on substrate saturation and the concept that at ^Inv^V_max_, each available site on the surface of a substrate, the so-called “attack sites”, is occupied by an enzyme molecule. Therefore, given two assays with identical substrates and identical surface areas, ^Inv^V_max_ can be seen as a measure of the intrinsic rate at which the enzyme degrades the substrate. The concentration of the enzyme at half-saturation with respect to the attack sites ^Inv^K_m_ can be interpreted similarly to that of the conventional K_m_ as a measure for substrate affinity. As the number of attack sites cannot be easily determined, neither ^Inv^K_m_ nor ^Inv^V_max_ can be determined in absolute terms for a particular substrate; however, different enzymes can be compared under, as best as possible, identical conditions.

**Figure 3:**
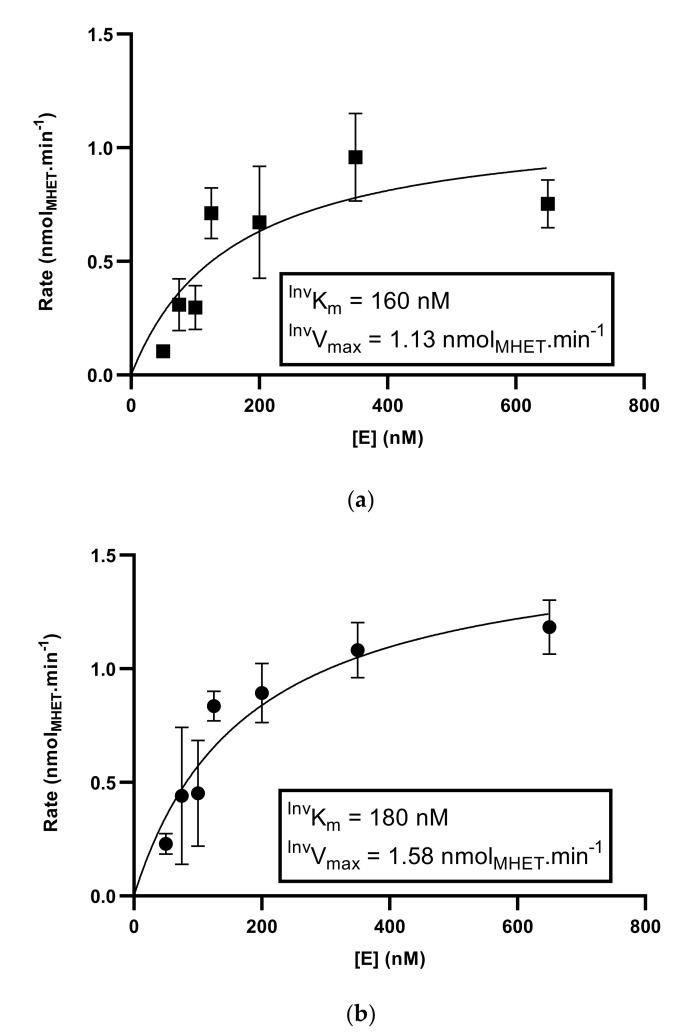
Determination of ^Inv^Km and ^Inv^Vmax for ^S136E^PETase (panel (**a**) and ^Son^PETase (panel (**b**)) after 6 h of hydrolysis of a standardised PET disc at 30 °C. The major product found by a concurrent HPLC analysis in all kinetic determinations was MHET, and given that the extinction coefficients of TPA and MHET are similar, concentrations are reported as MHET equivalents. Experimental values are shown as points on each graph. Error bars represent the standard deviations of triplicate measurements, and the line of best fit is fit to a Michaelis−Menten equation [15].

We thus compared the ^S136E^PETase with ^Son^PETase using the same substrate and assay conditions in order to compare the best performing variant in the current study with the previously described enzyme. The two curves are shown in Figure 3; See the Supplementary Materials for typical chromatograms showing MHET as the major product (S5).

Given that these kinetic hydrolysis assessments were also performed in the linear section of the progress curve for the reactions (See Supplementary material, S6), these can be considered as a measure of the intrinsic activity of the enzymes. The two values of ^Inv^V_max_ for ^S136E^PETase and ^Son^PETase calculated in these assays were 1.13 nmol_MHET_.min^−^^1^ and 1.58 nmol_MHET_.min^−^^1^, respectively, which are similar within the 95 % confidence intervals for the values calculated over all data points. The final point on the ^S136E^PETase curve appears to be a slight outlier, which pulls the calculated ^Inv^V_max_ value down, suggesting that the actual values of the two variants could be closer. Likewise, the ^Inv^K_m_ values for the two enzymes are very close, indicating a similar substrate affinity.

Based on this, we can conclude that the ^S136E^PETase variant performs at a similar level as ^Son^PETase, an enzyme that has been previously described as a promising variant for PET degradation [11]. Furthermore, this experiment has demonstrated that the apparent increase in the activity of the ^S136E^PETase variant relative to the wild-type enzyme, which is seen in Figure 2, is due to a genuine increase in the ability of the enzyme to degrade PET rather than the associated increase to thermostability. We therefore suggest that S136E is an interesting addition to the growing number of substitutions in IsPETase, which should be incorporated into further studies.

## Figures and Tables

**Table 1:** Alignment of mutations suggested by the PROSS server and confirmed by simulation, with the three thermostable PET hydrolases Tfcut2, LCC, and Cut190. Three of the mutations showed matches with the thermostable PET hydrolases and were experimentally produced and assessed for their thermostability and PET hydrolytic activity. Bold-labelled amino acids show agreements with the substitution suggested by PROSS; underlines are similar amino acids to those suggested by PROSS, while those that are italic are substitutions for an amino acid with a side chain of significantly different properties.

Substitution Designed by PROSS	S125R	S136E	T270Q
*Is*PETase	*S*	*S*	*T*
*Tfcut2*	*E*	N	**Q**
*LCC*	*S*	*T*	**Q**
*Cut190*	**R**	**E**	**Q**

## Data Availability

All pertinent data is included in the manuscript and supporting information.

## Supplementary Materials

The following are available online at https://www.mdpi.com/article/10.3390/polym13223884/s1: S1, Primers used during the study; S2, Full list of PROSS results; S3, Visualization of substitutions; S4, Thermal deactivation assay data; S5, Example chromatograms; S6, Inverse Michaelis–Menten progress curves.
