# Supplementary material for: Rational Protein Engineering to Increase the Activity and Stability of IsPETase Using the PROSS Algorithm
Source: Polymers (Basel). 2021 Nov 10;13(22):3884. doi: 10.3390/polym13223884 (PMC8621346; doi:10.3390/polym13223884)
Supplement: Supplementary file 1 [file polymers-13-03884-s001.zip › polymers-1452098-supplementary.pdf]

Supporting information

S1–Primers used during the study

**Function**	**Sequence (lowercase is mutant codon introduction)**
**SigPep Removal**	5’-ATATGCAGACCAATCCGTATGC-3’
	5’-TGAAATTCTTCCTCTATATG-3’
**T270Q**	5’-CCGTTACTCAcagTTCGCCTGTGAGAATC-3’
	5’-GTGTCATTATCCATGAATCG-3’
**S125R**	5’-CAGCCGTAGCcgcCAACAGATGGC-3’
	5’-CTGGGCTGGTCTAGAGTG-3’
**S136E**	5’-TCAAGTTGCGgaaTTGAACGGGACCAGCAGTAG-3’
	5’-CGAAGCGCGGCCATCTGT-3’

S2–Full list of PROSS results

	Wild Type	6QGC_design_1	6QGC_design_2	6QGC_design_3	6QGC_design_4	6QGC_design_5	6QGC_design_6	6QGC_design_7	6QGC_design_8	6QGC_design_9
40	A									P
254	G									A
208	I									V
94	I									V
190	N									D
225	N									T
193	S									G
213	S									T
223	S									T
245	S									D
46	S									P
189	T									Q
33	A								E	
117	L								Y	Y
172	N								D	D
173	N								R	R
224	R								Q	Q
280	R								S	S
166	S								A	A
169	S								A	A
116	T								L	L
279	T								S	S
180	A							I	I	I
248	A							D	D	D
65	A							G	G	G
182	Q							L	L	M
121	S							E	D	D
58	S							P	P	P
135	A						I	I	I	I
220	D						N	N	N	N
168	I						R	R	R	R
95	K						A	A	A	A
262	M						V	V	V	V
207	S						T	T	T	T
51	T						A	A	A	A
77	T						P	P	P	P
90	R					T	T	T	T	T
124	S					A	A	A	A	A
136	S					E	D	A	A	A
128	M				L	L	L	L	L	L
187	S				P	P	P	P	P	P
37	N			D	D	D	D	D	D	D
125	S			R	R	D	D	R	R	R
152	A		S	S	S	S	S	S	S	S
139	G		N	N	N	N	N	N	N	N
132	R		D	D	D	D	D	D	D	D
47	A	R	R	R	R	R	R	R	R	R
186	D	H	H	H	H	H	H	H	H	H
214	S	H	H	H	H	H	H	H	H	H
270	T	Q	Q	Q	Q	Q	Q	Q	Q	Q
134	V	L	L	L	L	L	L	L	L	L

S3–Visualisation of substitutions

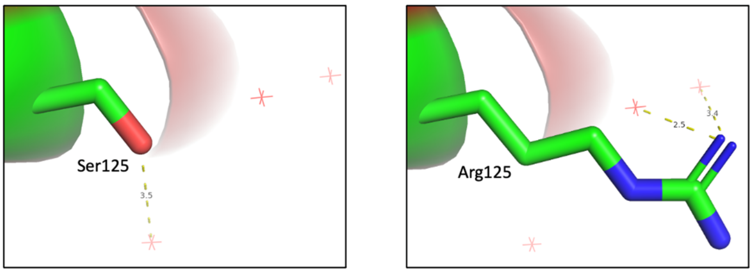

*Is*PETase ^{S125R}PETase

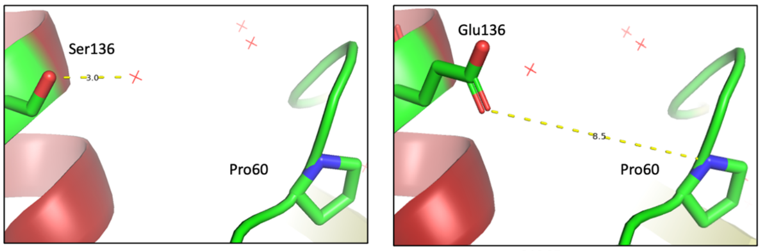

*Is*PETase ^{S136E}PETase

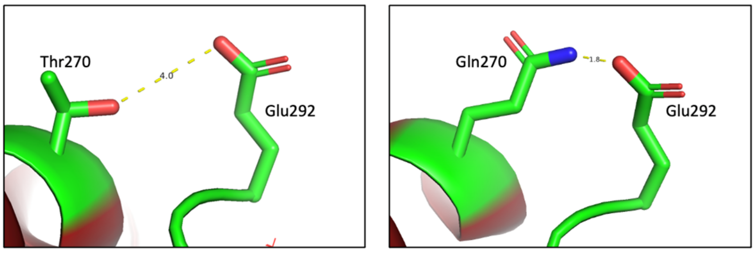

*Is*PETase ^{T270Q}PETase

S4–Thermal deactivation assay

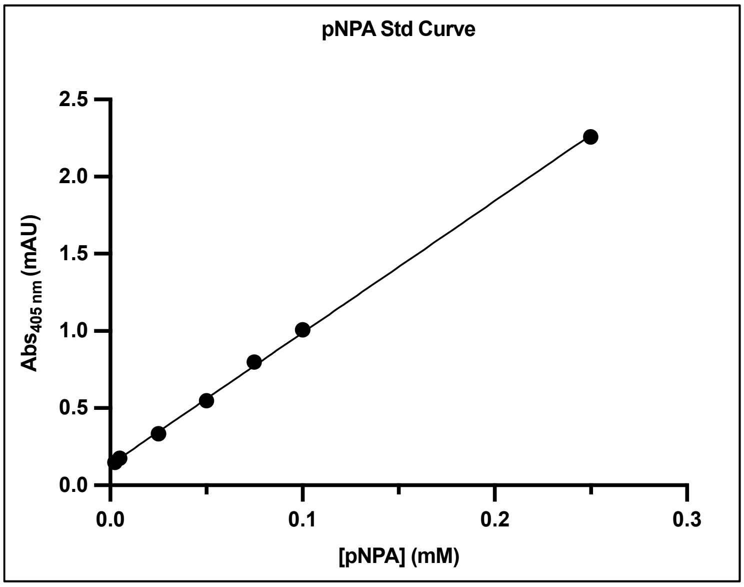

Linearity of pNPA with absorbance

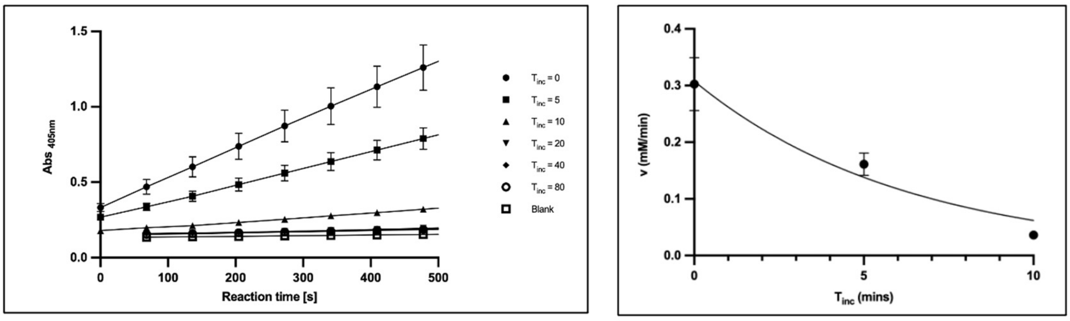

Wild-type enzyme thermal deactivation

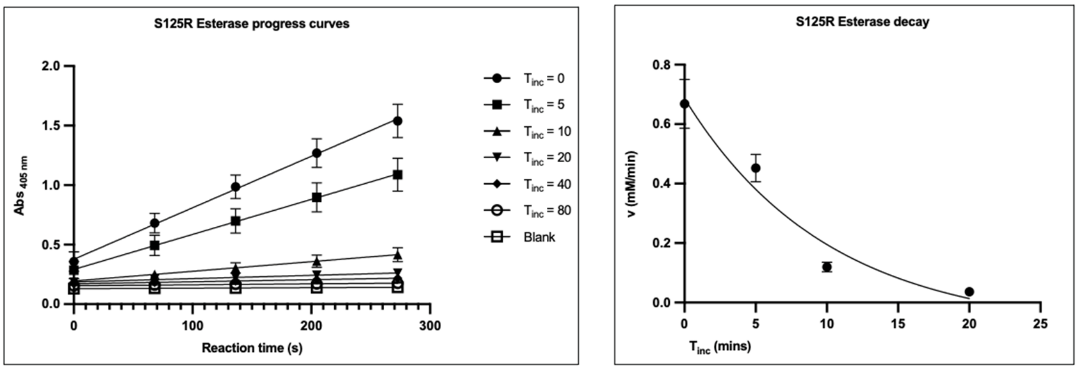

S125R PETase thermal deactivation

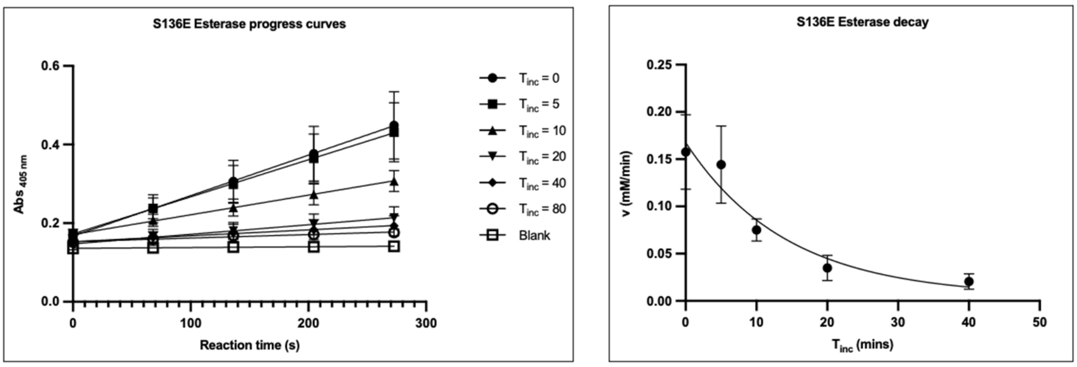

S136E PETase thermal deactivation

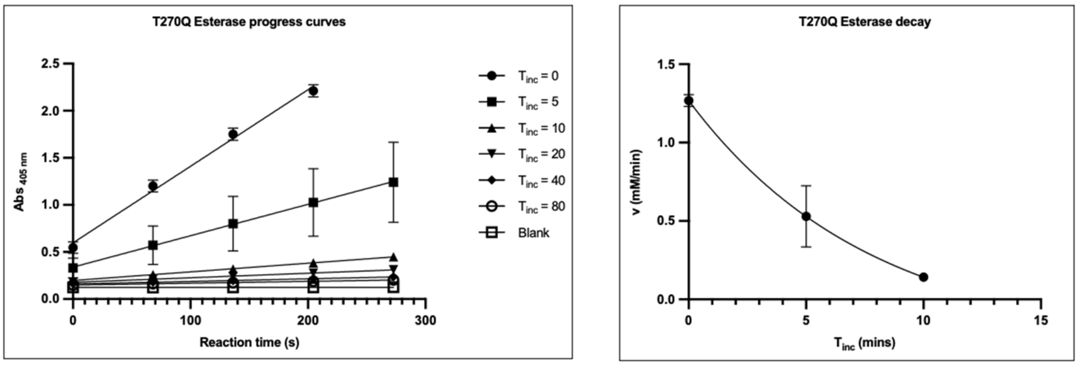

T270Q PETase thermal deactivation

S5–Example chromatograms

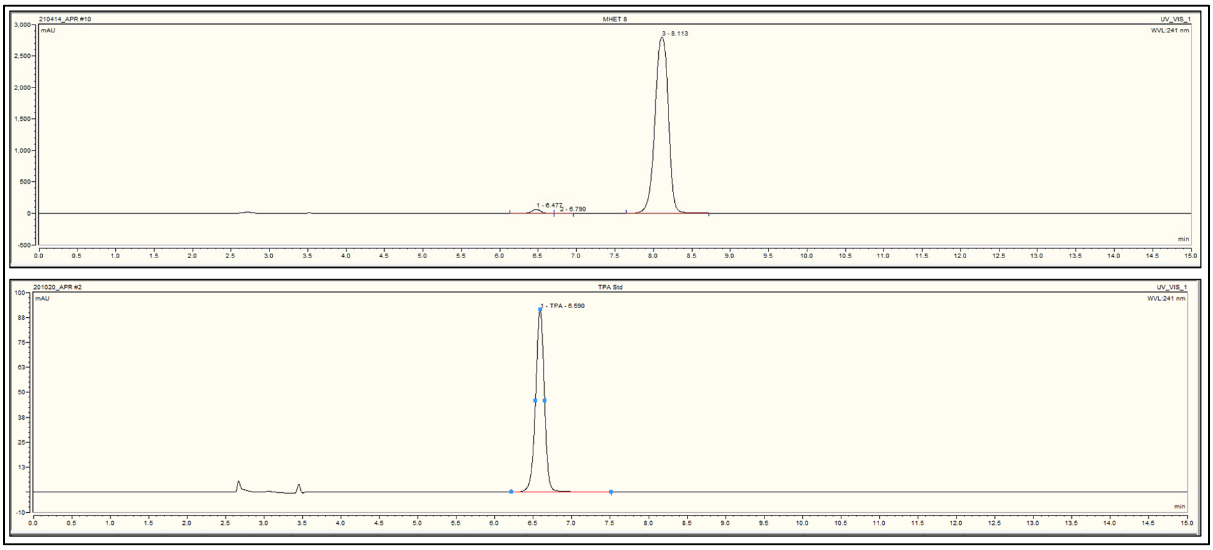

Example HPLC chromatograms of MHET standard (Upper panel) and TPA standard (Lower panel)

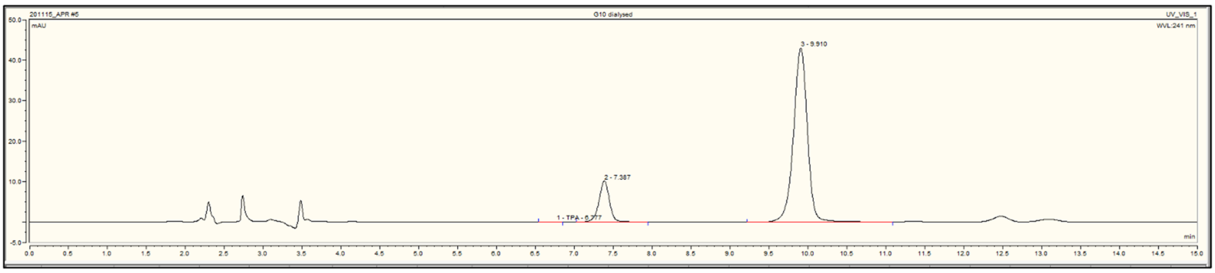

Typical HPLC chromatogram example, showing majority of degradation products are MHET. This was taken from the hydrolysis of PET powder experiment in which the active fractions of the gravity flow purification were identified. The small shift seen here relative to the standards is likely due to a different preparation of mobile phase. All peaks were identified to standards within the same run.

S6–^{Inv}MM progress curves

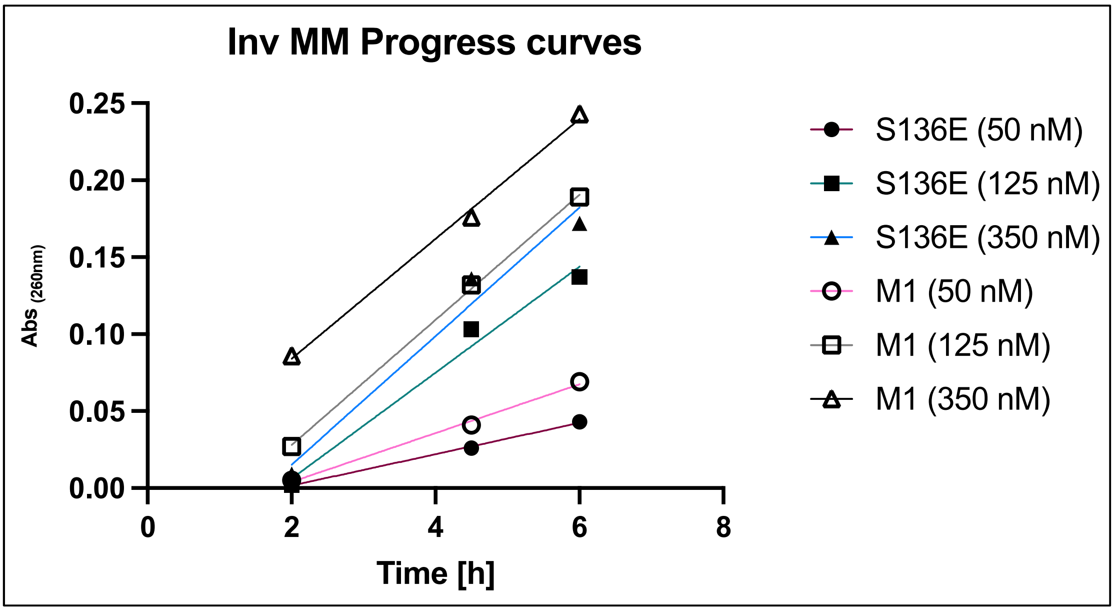

Progress curves for Inverse Michaelis Menten analyses
